# Factors associated with palliative care symptoms in cancer patients in Palestine

**DOI:** 10.1038/s41598-023-43469-0

**Published:** 2023-09-27

**Authors:** Maher Battat, Nawal Omair, Mohammad A. WildAli, Aidah Alkaissi, Husam T. Salameh, Riad Amer, Amer A. Koni, Sa’ed H. Zyoud

**Affiliations:** 1 Bone Marrow Transplant and Leukemia Unit, An-Najah National University Hospital, Nablus, 44839 Palestine; 2 Department of Hematology and Oncology, An-Najah National University Hospital, Nablus, 44839 Palestine; 3 Department of Nursing, College of Medicine and Health Sciences, An-Najah National University, Nablus, 44839 Palestine; 4 Department of Medicine, College of Medicine and Health Sciences, An-Najah National University, Nablus, 44839 Palestine; 5 Division of Clinical Pharmacy, Department of Hematology and Oncology, An-Najah National University Hospital, Nablus, 44839 Palestine; 6 Department of Clinical and Community Pharmacy, College of Medicine and Health Sciences, An-Najah National University, Nablus, 44839 Palestine; 7 Poison Control and Drug Information Center (PCDIC), College of Medicine and Health Sciences, An-Najah National University, Nablus, 44839 Palestine; 8 Clinical Research Center, An-Najah National University Hospital, Nablus, 44839 Palestine

**Keywords:** Health care, Oncology

## Abstract

Palliative care is critical to redundancy in cancer patients seeking to improve their quality of life. Evaluation should be incorporated into clinical practice routines at all stages of cancer. The Edmonton Symptom Assessment System (ESAS) was used to rate the intensity of ten symptom evaluations designed and validated for cancer patients in various languages and cultures. Therefore, the study aims to assess the symptoms reported using ESAS scores to identify patients who would benefit from palliative care that can improve the integration of palliative care into standard cancer care at An-Najah National University Hospital (NNUH). A cross-sectional study was selected for 271 cancer patients using a convenience sampling method at NNUH. Demographic, clinical, and lifestyle characteristics are described. Furthermore, patients' moderate to severe symptoms (score > 4) were obtained using ESAS-R. The survey consisted of 271 patients, with a response rate of 95%. The average age of the patients was 47 ± 17.7 years, ranging from 18 to 84 years. The male-to-female ratio was approximately 1:1, 59.4% of the patients were outpatients, and 153 (56.5%) had hematologic malignancies. Fatigue (62.7%) and drowsiness (61.6%) were the most common moderate to severe symptoms in ESAS. Furthermore, pain (54.6%), nausea (40.2%), lack of appetite (55.0%), shortness of breath (28.5%), depression (40.6%), anxiety (47.2%) and poor well-being (56.5%) were reported. In conclusion, fatigue and drowsiness were the most reported symptoms according to the ESAS scale among cancer patients, while moderate to severe symptoms were reported in cancer patients using the ESAS. The ESAS is a functional tool for assessing cancer patients' symptoms and establishing palliative care services.

## Introduction

Cancer is the second leading cause of death worldwide^1^ and the second leading cause of mortality in Palestine, accounting for 14% of all deaths, trailing only heart disease (30%)^2^. Furthermore, the number of Palestinians diagnosed with cancer is expected to increase, further reducing the financial and infrastructure resources of the current healthcare system, which are exacerbated by financial and political uncertainty^3^. Cancer, also known as malignancy, is abnormal cell growth. Solid tumors (breast, lung, skin, colon, and prostate cancer) and hematologic malignancies (such as lymphomas and leukemia) are among more than 100 types; symptoms vary depending on type^4^.

As effective cancer treatments continue to be discovered and refined, more patients are being cured, their life expectancy is extended, and more attention is paid to the psychological problems accompanying cancer diagnosis and treatment. According to studies, approximately 30% of patients have mental disorders^5^. Depression, for example, has a prevalence ranging from 1.5% to more than 53%^6^. Improved psychosocial and emotional well-being can be attributed to depression treatment and improved quality of life (QOL). This part of palliative care or supportive care can be used to treat cancer patients' physical and/or psychological symptoms. Palliative care is an important cancer care and treatment component that aims to slow, stop, or cure the disease^7^. It influences the physical, emotional, and psychological well-being of cancer patients^8^, which can start when diagnosed with cancer and may continue after cancer treatment^9^. Methods have been developed to assess the effectiveness of symptom management to help identify associated symptoms. The therapeutic aim of these instruments ranges from complete symptom and functional evaluation to in-depth examinations of particular symptoms. A tool devised and validated for rapid identification and monitoring of symptoms with minimal patient burden is the Edmonton Symptom Assessment System (ESAS). The ESAS was developed to help assess pain, fatigue, nausea, depression, anxiety, drowsiness, appetite, well-being, and shortness of breath (SOB) among cancer patients^10^. From the review of the literature, the following studies used the ESAS-R: As reported in a prospective observational study using the Arabic version, the most common severe symptoms among Egyptian cancer patients were pain (93%), followed by fatigue (74%), poor health (67%), lack of appetite (62%), anxiety (60%) and drowsiness (56%)^11^. Furthermore, an international multicenter observational study (European Palliative Care Research Collaborative—Computerized Symptom Assessment and Classification of Pain, Depression, and Physical Function) was conducted in 2008 and 2009 to assess depression among 1051 cancer patients recruited from 17 centers in eight countries; 696 patients completed an evaluation of depression using the ESAS and Patient Health Questionnaire-9 [PHQ-9]. This study aimed to investigate the association between self-reported depression disorder (DD) and symptoms in patients with advanced cancer controlled for prognostic factors. Grotmol concluded that depression in advanced cancer patients causes a high burden of symptoms, which affects the patient's somatic symptoms. Using ESAS to identify depression in cancer patients and treat it is critical in palliative care to improve patient quality of life^12^. A retrospective study in the United States of America reviewed the charts of 216 patients; the instruments used the ESAS and the subscales of the hospital anxiety and depression scale (HADS-A and HADS-D) to determine the relationship between the frequency and intensity of physical symptoms and their expression of depression and anxiety. Delgado-Guay et al. concluded that the frequency and intensity of the expression of physical and psychological symptoms vary in patients with advanced cancer with depression. A remarkable connection was observed between the presentation of depression and the expression of mental well-being and psychic symptoms. In addition, the detection of mood disorders must be performed, or patients with high expression and/or intensity of multiple symptoms should implement screening protocols in outpatient and hospital settings for early detection and management of untreated physical symptoms and psychological abnormalities using simple tools such as ESAS and BDI II-21^13^. In Poland, a cross-sectional study was conducted among 800 cancer patients of the Podkarpackie Cancer Center, Clinical Provincial Hospital in Rzeszów in 2018–2020 who received chemotherapy. The purpose of the study was to evaluate the quality of life. One of the instruments used was the ESAS, which concluded that cancer undoubtedly harms the patient’s quality of life, which is related to the disease process, the treatment used, and the disease duration as reported symptoms of anxiety and depression^14^.

However, Jordan was among the first Arab countries to be accredited in this domain^15^. This concept had not previously been studied in Palestinian cancer patients. Therefore, this study aims to assess palliative care symptoms in the Palestinian cancer population, determine the associations between demographic and clinical characteristics of patients with each ESAS domain, and find predictors of ESAS.

This study aims to introduce a palliative care assessment tool within the Palestinian population, thus contributing to the foundation of palliative care principles in Palestine. In addition, it describes a 'new' population quite different from many populations described in Europe, the Americas, and Asia. In addition, it provides substantiating evidence for addressing the physical symptoms and their effects on the quality of life in patients experiencing palliative cancer. Furthermore, it determines whether the subsequent intervention will benefit palliative care in cancer patients and whether it is possible to identify cases with acceptable sensitivity and specificity. The utility is also more relevant when ‘non-specialists’ can perform the screening with an instrument in the subject under consideration that is easy to use in routine clinical practice. Finally, increased recognition in the detection and treatment of comorbid depression in hospitals also improves the management of symptoms.

## Methods

### Study design

A cross-sectional study was conducted to achieve the objectives of the study.

### Study setting

The An-Najah National University Hospital (NNUH) is in Nablus' northern mountainous region, near the Asira Al Shamaliah exit. Established in 2013, NNUH is composed of 120 beds. The hospital provides services for cancer treatments, including surgeries, chemotherapy, biological therapy, autologous bone marrow transplant, and other departments with many other advanced and modern services. The hospital is considered the main referral center for hematologic malignancies, and it is the only center in Palestine that provides autologous bone marrow transplantation for multiple myeloma, Hodgkin lymphoma, and non-Hodgkin lymphoma.

### Study population

Cancer patients in NNUH consult outpatient oncology clinics and receive treatment in outpatient oncology clinics. Inpatients may come for diagnosis, chemotherapy cycle, and autologous bone marrow transplant or treatment of side effects/complications.

### Sample size

The NNUH was visited by approximately 600 cancer patients monthly during the study period (April 2021–August 2021). This population size was used to determine the sample size needed for the analysis. A sample size of 235 was calculated using the Raosoft sample size calculator by setting the response distribution at 0.50, the error margin at 5%, and the confidence interval at 95%. When we calculated using Raosoft, 259 patients were needed to cover the dropout. Therefore, we added 10% of the sample (24 patients), and the target sample size increased to 285 participants to decrease erroneous results and improve the reliability of the research.

The pilot test was conducted first for 10% of the sample size (24 questionnaires). It was excluded from the study because sociodemographic data was edited after the content validity and the reliability of the internal consistency of the questionnaire were tested. The validity of the data was tested for their content only by triangulation, a panel that included hematologists, oncologists, three oncology nurses, and one statistician. The reliability was assessed for 11 patients (22 questionnaires) between two visits. Furthermore, after developing the questionnaire, the content and design were tested in a pilot in 11 patients, with modifications made as needed. Only some questions were modified to be clearer within categories and easier to respond to without writing, so the questionnaire was completed in less time, and some of the duplicate variables, prognostic factors, stages, and palliative care were removed.

### Sampling procedure

The convenience sampling method consisted of 271 cancer patients.

### Inclusion and exclusion criteria

Inclusion criteria:

1. Patients who agreed to participate
2. Individuals 18 years and older can read and write.
3. Both sexes
4. Inpatients and outpatients with cancer and hematologic malignancies.

Exclusion criteria:

1. Patients who need ICU care.
2. Comatose patients.
3. Patients with cognitive impairment.
4. Patients in isolation.

### Data collection instrument

The palliative care symptoms ESAS-R is valid and reliable for assessing nine common symptoms common to cancer patients^16^. This tool assesses pain, fatigue, nausea, depression, anxiety, drowsiness, appetite, well-being, and SOB. Each patient was provided a blank carbon form with the ESAS-R questionnaire. Oncology nurses guided as needed to facilitate the completion of the forms. Patients can use a blank scale to assess "other problems" as needed. Furthermore, a numerical scale of 0 to 10 is used to rate the severity of each symptom, with 0 denoting the absence of the symptom and 10 being the worst possible severity^17^. The ESAS is a useful screening tool to assess psychological symptoms, including depression, which is easy to use by cancer care team professionals to provide the necessary palliative care and regular evaluation of the patient using the ESAS and a cutoff point > 3^18^.

The data was collected over five months, from April 2021 to August 2021. This data was collected through cross-sectional observations at various points during cancer treatment, encompassing the periods of diagnosis, chemotherapy, clinic visits, and autologous bone marrow transplantation (Auto-BMT). These observations were conducted for patients in advanced stages of cancer, both in outpatient and inpatient oncology settings. The researcher documented this information in separate papers. The Arabic version^19^ was immediately provided to the patient directly by the delegated researcher or nurse (one nurse in outpatient oncology clinics and one nurse on the medical oncology ward), and all questionnaires were completed by the patients or read to them by the researcher or the two delegated nurses. If the patients had difficulty understanding the question's meaning, we explained it simply. All instruments were completed in paper forms and the questionnaires were saved in a special file in the targeted wards that receive adult patients with oncologic and/or hematologic malignancies: outpatient oncology clinics, medical oncology ward, vascular ward, surgical ward, bone marrow transplant and leukemia ward, and surgical cardiac care unit. Then, other medical-related information taken from patient files by the researcher was entered into an electronic database for analysis. Demographic data from patients and clinical factors were also collected. Approximately 15 patients refused to participate and ten incomplete questionnaires were excluded.

### Statistical analysis

The Statistical Package for Social Sciences (SPSS) version 21 was used to enter and analyze the data. Descriptive statistics such as frequencies, percentages, means (standard deviations), and medians (interquartile ranges) were used to summarize basic demographic information. The Mann‒Whitney U and Kruskal‒Wallis tests were used to determine the association between independent variables and ESAS scores. All statistical tests were two-sided, with P values less than 0.05 considered statistically significant. In the bivariate analysis, all variables that showed a significant correlation with ESAS scores, including sociodemographic and clinical factors, were included in a multiple linear regression model. This model was used to identify the most important variables associated with each dimension of ESAS.

### Ethics approval and consent to participate

Institutional Review Boards (IRBs) and local health authorities approved all components of the study protocol, including access to and use of patient clinical data, IRB approval no. (Mas. Feb. 2021/17), in which the human body is protected with no risk. This study was carried out following the Helsinki Declaration, and European guidelines for good clinical practice and approval were requested and obtained from the NNUH search center. We confirm that the information collected was used only for clinical research. All personal information provided by the patients is kept private and used only for this study. All participants received an informed consent form that confirmed data privacy, and all data were kept confidential and used specifically for research purposes. All information was stored in a locked cabinet for human body rights, and there was no access to anyone except the researcher. The IRB of An-Najah National University approved only verbal consent. The reason for verbal consent is that participants were only required for the interview and were not subjected to any harm as long as their privacy was kept confidential. The authors confirmed that all the methods were performed following the relevant guidelines and regulations.

## Results

### Demographic data

A total of 271 patients were included in the study, with a response rate of 95%. Fifty-two percent were 50 years or older and most participants (n = 184, 67.9%) were married. The demographics of the patients are summarized in Table 1. The mean age of the patients was 47 ± 17.7 years, with a range of 18–84 years. The male to female ratio was approximately 1:1 (51.3% and 48.7%, respectively). Regarding education, the majority of the participants (n = 183, 67.5%) went to school, and 88 (32.5%) completed university or college. The socioeconomic status of the subjects was as follows: 146 (53.9%) were affordable with low income, 104 (38.4%) were good and only 21 (7.7%) were very good. Among all participants, 13 (4.8%) had deformities such as Tal Hashomer syndrome, 60 (22.1%) were smokers, 99 (36.5%) had a job, 111 (41.0%) lived in a city and 129 (47.6%) lived in a village. Moreover, 59.4% of the individuals were treated as outpatients, and among them, 56.5% received a diagnosis related to hematologic malignancies. These hematologic malignancies included acute lymphoblastic leukemia (ALL) at 9.6%, acute myeloid leukemia (AML) at 10.3%, chronic lymphocytic leukemia (CLL) at 2.2%, Hodgkin lymphoma (HL) at 11.8%, non-Hodgkin lymphoma (NHL) at 11.4%, multiple myeloma (MM) at 9.6%, myelodysplastic syndromes (MDS) at 1.5%. On the other hand, 43.5% of patients were diagnosed with solid tumors, which encompassed various types, including breast cancer at 14.0%, colorectal cancer at 6.6%, gastric cancer at 3.0%, duodenal cancer at 0.7%, pelvic retroperitoneal mass at 0.4%, sarcoma at 3.3%, uterine cancer at 1.1%, ovarian cancer at 1.8%, teratoma at 0.4%, bladder cancer at 1.8%, pancreatic cancer at 3.7%, gallbladder cancer at 0.7%, lung cancer at 2.2%, hepatocellular carcinoma at 0.4%, nasopharyngeal cancer at 0.4%, vocal cord cancer at 0.4%, larynx cancer at 0.4%, prostate cancer at 1.1%, malignant mesothelioma at 0.4%, esophageal cancer at 0.4%, and brain cancer at 0.4%. Some participants had a history of comorbid diseases, such as hypertension (24.4%), diabetes mellitus (18.1%), respiratory problems such as asthma (9.6%), Crohn's disease (0.7%), end-stage renal disease (ESRD) (1.1%), and gout (1.8%), and bone-related problems such as osteoporosis (10.7%), neurologic problems (5.7%), genitourinary disorders (5.2%), ophthalmic problems (18.8%), hyperthyroidism (0.7%), hypothyroidism (0.4%), liver cirrhosis (0.4%), and rheumatism (0.7%). In particular, most cancer patients (n = 241, 88.9%) were in the treatment stage and 205 (75.6%) were actively on the chemotherapy protocol. Regarding the types of support patients received, family psychological support was the main type (59.8%). 44.3% received support from the healthcare team, 38.0% received religious support, and 34.3% received social support.

**Table 1:** Characteristics of the patients.

Variable	Frequency (%)
Age	
≤ 50	141 (52.0)
> 50	130 (48.0)
Gender	
Male	139 (51.3)
Female	132 (48.7)
Marital status	
Singe	87 (32.1)
Married	184 (67.9)
Educational level	
School	183 (67.5)
University or college	88 (32.5)
Socioeconomic status	
Affordable (low income)	146 (53.9)
Good (middle income)	104 (38.4)
Very good (high income)	21 (7.7)
Deformities	
Yes	13 (4.8)
No	258 (95.2)
Smoker	
Yes	60 (22.1)
No	211 (77.9)
Work	
Yes	99 (36.5)
No	172 (63.5)
Living location	
City	111 (41.0)
Village	129 (47.6)
Camp or refugee	31 (11.4)
Hospitalization status	
Inpatient	110 (40.6)
Outpatient	161 (59.4)
Type of cancer	
Hematology	153 (56.5)
Solid	118 (43.5)
Treatment stage	
Yes	241 (88.9)
No	30 (11.1)
Currently on chemotherapy	
Yes	205 (75.6)
No	66 (24.4)
Recently, pancytopenia	
Yes	86 (31.7)
No	185 (68.3)
Autologous bone marrow transplant (auto-BMT)	
Yes	24 (8.9)
No	247 (91.1)
Admitted for surgery	
Yes	10 (3.7)
No	261 (96.3)
Types of psychological support	
Family support	162 (59.8)
Social support	93 (34.3)
Religious support	103 (38.0)
Health care team support	120 (44.3)

### ESAS symptoms

Table 2 shows ESAS symptoms among study participants. Current findings reported that the mean fatigue score (fatigue) was 4.6 ± 3.0, and 62.7% of the patients complained of moderate to severe tiredness. Furthermore, 61.6% had moderate to severe drowsiness, with a mean score of 4.5 ± 3.0. The frequency of other moderate to severe symptoms was the following: pain (54.6%), nausea (40.2%), loss of appetite (55.0%), SOB (28.4%), depression (40.6%), and anxiety (47.2%).

**Table 2:** Description of ESAS symptoms.

ESAS symptoms	Mean ± SD	Median [Q1–Q3]	Frequency (%) of moderate to severe symptoms
Pain	4.1 ± 3.1	4.0 (1.0–6.0)	148 (54.6)
Tiredness	4.6 ± 3.0	5.0 (2.0–7.0)	170 (62.7)
Drowsiness	4.5 ± 3.0	5.0 (2.0–7.0)	167 (61.6)
Nausea	3.1 ± 3.1	2.0 (0.0–5.0)	109 (40.2)
Appetite loss	4.1 ± 3.2	4.0 (1.0–7.0)	149 (55.0)
SOB (SOB)	2.2 ± 2.7	1.0 (0.0–4.0)	77 (28.4)
Depression	3.2 ± 3.0	2.0 (1.0–5.0)	110 (40.6)
Anxiety	3.8 ± 3.2	3.0 (1.0–6.0)	128 (47.2)
Wellbeing	4.0 ± 3.1	4.0 (1.0–6.0)	153 (56.5)

Regarding the well-being dimension in the ESAS, the highest value means the worst feeling of well-being, and the frequency of moderate to severe feelings of poor well-being was 56.5%, with a mean score of 4.0 ± 3.1

### The total ESAS score

The total score of the ESAS indicated the overall burden of symptoms, as shown in Table 3, which does not show a significant relationship between the total score of the ESAS and sociodemographic data.

**Table 3:** Total score dimension (ESAS).

Variable	Total score Median [Q1–Q3]	P value
Age		
≤ 50	184 [110.0–245.0]	0.267
> 50	161 [93.7–236.3]	
Gender		
Male	170.0 [111.0–230.0]	0.717
Female	166.0 [93.3–256.3]	
Marital status		
Single	173.0 [111.0–247.0]	0.450
Married	165.0 [94.5.0–234.0]	
Educational level		
School	167.0 [104.0–234.0]	0.611
University or college	177.0 [101.0–257.0]	
Socioeconomic status		
Affordable (low)	180.0 [103.7–245.5]	0.1
Good (middle)	167.0 [105.5–232.0]	
Very good (high)	123.0 [69.5–170.0]	
Deformities		
Yes	170.0 [1.01.5–264.0]	0.703
No	238.5 [168.0–277.0]	
Smoker		
Yes	183.0 [108.0–239.5]	0.285
No	166.0 [101.0–240.0]	
Work		
Yes	179.0 [106.0–232.0]	0.553
No	165.5 [96.5–273.0]	
Living location		
City	178.0 [105.0–240.0]	0.39
Village	159.0 [93.5–234.5]	
Camp or refugee	210.0 [119.0–257.0]	
Hospitalization status		
Inpatient	181.0 [79.3–255.3]	0.935
Outpatient	167.0 [109.5–228.0]	
Type of cancer		
Hematology	183 [92.0–251.0]	0.392
Solid	160.5 [105.7–116.7]	
Treatment stage		
Yes	169.0 [108.0–239.0]	0.214
No	134.5 [61.3–252.3]	
Currently on chemotherapy		
Yes	165.0 [98.0–240.0]	0.602
No	169.5 [107.3–236.7]	
Recently, pancytopenia		
Yes	166.0 [86.7–249.0]	0.442
No	170.0 [108.5–236.5]	
AutoBMT		
Yes	202.0 [90–228.5]	0.97
No	167.0 [104.0–244.0]	
Admitted for surgery		
Yes	144.5 [64.0–211.0]	0.3
No	169.0 [104.5–242.0]	
Types of psychological support		
Family support	Yes 169.5 [99.5–246.0]	0.799
	No 167.0 [104.5–231.0]	
Social support	Yes 169.0 [96.0–249.5]	0.701
	No 168.0 [104.7–232.7]	
Religious support	Yes 173.0 [106.0–251.0]	0.264
	No 165.5 [101.0–232.0]	
Health care team support	Yes 185.0 [106.3–250.5]	0.238
	No 164.0 [101.0–232.0]	

### Pain dimension

According to ESAS, the dimension of pain was significantly associated with many factors, as shown in Additional file 1: Table S1, including age (*p* = 0.003), sex (*p* = 0.007), marital status (*p* = 0.001), stage of work (*p* = 0.001), stage of treatment (*p* = 0.039), chemotherapy (*p* = 0.036), type of cancer (*p* < 0.001), and pancytopenia condition (p = 0.011).

First, we found that cancer patients aged > 50 years had more pain. The median score [Q1-Q3] was 4.0 [2.0–7.0] compared to those aged < 50 years, with a median [Q1-Q3]: 3.0 [1.0–5.0]. It was also found that women had a higher pain score than men. Furthermore, pain was higher in cancer patients in the treatment stage, with a score of 4.0 [1.0–6.0], than in cancer patients in the diagnosis stage, with a score of 3.0 [0.7–5.0]. Cancer patients who underwent chemotherapy were reported to have significantly more pain, with a pain score of 4.0 [2.0–6.0], than those who did not actively receive chemotherapy. Furthermore, patients with solid tumors had significantly higher pain scores than patients with hematologic malignancies (*p* < 0.001). However, in the regression analysis, only work and type of cancer were significantly associated with the pain domain, as shown in Table 4.

**Table 4:** Multiple linear regression analysis for variables associated with ESAS symptoms.

Model	Unstandardized coefficients		Standardized coefficients	t	p value*	95.0% confidence interval for B		Collinearity statistics
	B	Std. error	Beta			Lower bound	Upper bound	VIF
Pain								
(Constant)	0.618	1.395		0.443	0.658	− 2.130	3.366	
Age	0.639	0.392	0.103	1.630	0.104	− 0.133	1.411	1.184
Gender	0.475	0.397	0.077	1.196	0.233	− 0.307	1.256	1.214
Marital status	0.113	0.283	0.025	0.401	0.689	− 0.444	0.670	1.163
Work	0.824	0.405	0.128	2.033	**0.043**	0.026	1.621	1.175
Type of cancer	1.021	0.455	0.164	2.242	**0.026**	0.124	1.917	1.572
Treatment stage	− 0.425	0.619	− 0.043	− 0.686	0.493	− 1.643	0.794	1.163
Currently on chemotherapy	− 0.653	0.433	− 0.091	− 1.507	0.133	− 1.507	0.201	1.068
Recently, pancytopenia	0.012	0.482	0.002	0.024	0.981	− 0.937	0.960	1.550
Tiredness								
(Constant)	2.073	0.866		2.394	0.017	0.368	3.779	
Marital status	0.035	0.264	0.008	0.131	0.896	− 0.486	0.555	0.957
Work	0.837	0.370	0.136	2.262	**0.025**	0.108	1.567	0.992
Type of cancer	0.734	0.367	0.123	2.000	**0.046**	0.011	1.456	0.954
Nausea								
(Constant)	5.304	0.689		7.693	0.000	3.946	6.661	
Socioeconomic status	− 0.715	0.290	− 0.148	− 2.468	**0.014**	− 1.285	− 0.144	0.992
Currently on chemotherapy	− 0.870	0.429	− 0.122	− 2.029	**0.043**	− 1.714	− 0.026	0.992
Appetite								
(Constant)	8.679	1.713		5.067	0.000	5.306	12.051	
Marital status	0.336	0.274	0.071	1.227	0.221	− 0.204	0.876	0.992
Socioeconomic status	− 0.679	0.302	− 0.133	− 2.245	**0.026**	− 1.274	− 0.083	0.952
Smoker	− 1.141	0.460	− 0.146	− 2.482	**0.014**	− 2.046	− 0.236	0.965
Work	1.043	0.395	0.155	2.644	**0.009**	0.266	1.820	0.974
Hospitalization	− 0.930	0.400	− 0.141	− 2.324	**0.021**	− 1.718	− 0.142	0.910
Auto_BMT	− 1.213	0.686	− 0.106	− 1.767	0.078	− 2.564	0.139	0.924
Shortness of breath								
(Constant)	3.107	0.868		3.580	0.000	1.399	4.816	
Smoker	− 0.936	0.389	− 0.145	− 2.403	**0.017**	− 1.703	− 0.169	0.997
Family support	0.537	0.330	0.098	1.629	0.105	− 0.112	1.186	0.997
Depression								
(Constant)	6.274	0.847		7.407	0.000	4.606	7.941	
Socioeconomic status	− 0.673	0.279	− 0.144	− 2.408	**0.017**	− 1.223	− 0.123	0.986
Smoker	− 1.165	0.427	− 0.163	− 2.727	**0.007**	− 2.007	− 0.324	0.986
Anxiety								
(Constant)	2.707	0.839		3.226	0.001	1.055	4.359	
Education	− 0.118	0.223	− 0.031	− 0.527	0.598	− 0.557	0.322	0.952
Socioeconomic status	− 0.966	0.301	− 0.191	− 3.211	**0.001**	− 1.559	− 0.374	0.953
Treatment Stage	2.518	0.596	0.246	4.227	** < 0.001**	1.345	3.690	0.997
Wellbeing								
(Constant)	9.947	1.920		5.180	0.000	6.167	13.728	
Socioeconomic status	− 0.754	0.284	− 0.157	− 2.654	**0.008**	− 1.313	− 0.195	0.980
Deformities	− 2.354	0.838	− 0.165	− 2.808	**0.005**	− 4.004	− 0.704	0.995
Smoker	− 0.958	0.436	− 0.130	− 2.196	**0.029**	− 1.817	− 0.099	0.973
Work	0.895	0.374	0.141	2.391	**0.017**	0.158	1.633	0.982

Significant values are in bold.

### Fatigue dimension

As indicated in Additional File 1: Table S2, fatigue among cancer patients was related to marital status (*p* = 0.006), and married patients experienced increased fatigue, which is associated with a high level of fatigue due to low income, as well as work (*p* = 0.042), which causes fatigue in non-workers and solid tumors (*p* = 0.021). We found work and type of cancer as predictors for the fatigue domain, as shown in Table 4.

### Drowsiness dimension

As shown in Additional file 1: Table S3, drowsiness is significantly associated with socioeconomic status (*p* = 0.002), which is high at the low-income level, with a median score of 5 from 10.

### Nausea dimension

Additional File: Table S4 shows that the nausea score was significantly higher in patients receiving active chemotherapy, with a score of 3.0 [1.0–5.0] and a *p-value of 0.023,* and was significantly associated with socioeconomic status (*p* = 0.007) and patients currently receiving chemotherapy for nausea, as predicted in the linear regression analysis in Table 4.

### Lack of appetite dimension

As shown in Additional file 1: Table S5, lack of appetite was significantly associated with *smokers* (*p* = 0.016), hospitalized patients (*p* = 0.007) and AutoBMT (*p* = 0.037) and was associated with marital status (*p* = 0.002), work, and socioeconomic status (*p* = 0.004). In the multiple linear regression analysis, we reported that hospitalized patients, socioeconomic status, smoking, and work were substantially associated with a lack of appetite, as shown in Table 4.

### Shortness of breath dimension

In Additional file 1: In Table S6 of the SOB domain, smoking was reported to be highly associated with this symptom (*p* = 0.021) and was the only predictor of SOB, as shown in Table 4, where the SOB score was 2.5 [0.0–5.0] for smokers and 1.0 [0.0–3.0] for non-smokers. Furthermore, SOB was significantly associated with family support (*p* = 0.021), indicating a lower severity of family support for SOB.

### Depression dimension

As shown in Additional file 1: In Table S7, variables such as smoking (*p* = 0.004) and good or affordable socioeconomic status (*p* = 0.026) were significantly associated with depression, as shown in Table 4.

### Anxiety dimension

As shown in Additional file 1: In Table S8, anxiety was found to be associated with educational level (*p* = 0.044) and treatment stage (*p* < 0.001). The anxiety score was 5.0 [3.8–9.3] for cancer patients in the diagnosis stage, while the score was 3.0 [1.0–6.0] for those in the treatment phase and was significantly associated with socioeconomic status (*p* = 0.012). However, two factors, socioeconomic status and treatment phase, were determinants of anxiety, as shown in the regression analysis in Table 4.

### Wellbeing dimension

In the current analysis, as shown in Additional file 1: In Table S9, a poor feeling of well-being was identified in cancer patients with deformities (*p* = 0.026), with a score of 5.0 [3.5–9.5], compared to cancer patients without deformities, 4.0 [1.0–6.0], and well-being was significantly associated with socioeconomic status (*p* = 0.016) and smoking (*p* = 0.028). Socioeconomic status, deformities, smoking, and work status are predictive well-being factors, as shown in Table 4.

## Discussion

This cross-sectional study carried out at NNUH in a developing country sheds light on the significant burden of symptoms experienced by cancer patients seeking palliative care. The current study used the ESAS to assess the intensity of various symptoms commonly encountered by cancer patients.

In our study sample, the male-to-female ratio was approximately 1:1, similar to Palestine's general distribution of malignancies. According to the Palestinian Ministry of Health Report^20^, in 2020, there were 49.3% male patients and 50.7% female patients. However, a similar study in Italy showed that 58% of the participants were women^18^.

In our study, the mean age of the participants was 47 years, while in other studies, the mean age was 49.12 years^21^ and 61.9 years^18^. In our study, 88.9% of cancer patients were in the treatment stage and the others were in the diagnostic stage. This percentage is similar to a previous study that included patients recently on a chemotherapy protocol (82%)^18^.

The most common symptoms reported by our study were fatigue (62.7%), drowsiness (61.6%), poor health (56.5%), loss of appetite (55.0%), and pain (54.6%). In a study conducted in Egypt, the symptoms of advanced cancer were pain (93%), followed by fatigue (74%), poor well-being (67%), lack of appetite (62%), anxiety (60%), and drowsiness (56%)^11^. However, another study reported that pain was the most common symptom in the diagnosis stage of incurable cancers^22^. Furthermore, most of the patients included in the study were tired (94%), anxious (87.5%), and depressed (83%)^23^. Using the ESAS scale as a guide to identify and understand the main problems can help establish appropriate care for cancer patients^24^.

In a study analyzed, the median (range) score for depression was 2 (0–10) in the ESAS, with a cutoff of 2 out of 10 or more, having a sensitivity of 77% and 83%, respectively, with a specificity of 55% and 47% for depression and moderate/severe depression^25^.

Unfortunately, there is a lack of specialized centers or palliative care specialists in Palestine, which is essential to reduce the intensity of these symptoms^26^. In the United States of America (USA), research has examined the determinants of symptom improvement in 406 advanced cancer patients referred to palliative care. In this study, fatigue was more likely to improve in individuals with higher levels of other symptoms at baseline, such as dyspnea, sadness, and nausea. Pain relief was more prevalent in drowsy patients. Old age was associated with better health after 1–4 weeks of palliative care^27^.

Similarly, Canadian researchers looked at the factors influencing improvement in 150 cancer patients who participated in a palliative care team intervention. This study found that after one week of intervention, female sex was related to improved symptoms, with nausea, anxiety, dyspnea, and pain showing the greatest improvement^28^. Another study in the USA examined gastrointestinal symptoms in 202 advanced cancer patients referred for palliative surgical consultation. Again, surgical treatment patients had better symptoms than those who did not, and there was no link between improvement and sex, age, or current chemotherapy or biotherapy^29^.

In the current analysis, women with cancer and those aged > 50 years were found to have significantly higher pain scores. It seems that older individuals with depression may be more likely to show discomfort due to concurrent health conditions^30^. Additionally, those who worked had lower pain scores, possibly because work requires the body to move, which is excellent for circulation, prevents muscular tightness and joint stiffness, and raises the pain tolerance threshold^31^. The pain score was significantly higher in cancer patients in the treatment stage. This could be due to the adverse effects of anticancer medications, such as chemotherapy-induced peripheral neuropathy (Vince alkaloid)^32^. Pain was more severe in solid tumors (median pain score = 4) than in hematologic malignancies (median pain score = 3), which is supported by previous studies^33^.

As reported in the present study, the fatigue score was lower in workers than in non-workers. It should be noted that fatigue due to malignancy is not alleviated by rest. This symptom is multifactorial, either the primary disease or the side effects of cancer therapy. However, the specific underlying pathophysiology is unknown^34,35^. As expected, cancer patients actively on chemotherapy had significantly higher nausea scores than those who did not. Nausea and vomiting are distressing symptoms. Despite the availability of strong antiemetic and evidence-based recommendations, up to 40% of cancer patients receiving chemotherapy experience nausea and vomiting^36^.

Furthermore, the SOB score was significantly higher in smokers than in non-smokers, consistent with previous findings that identified increased dyspnea in cancer patients who smoke^37^. Furthermore, the depression score showed a statistically significant association between socioeconomic status and smoking, supported by other studies^38–41^. Regarding the anxiety score associated with educational level, the anxiety level was higher in cancer patients with an educational level of school and lower educational level, with a score of 4.0. The anxiety score in patients with a university or college educational level was 2.0, perhaps because a higher education level appears to have a protective impact against the accumulation of anxiety and sadness throughout life^42^. Our results showed that anxiety was higher in cancer patients in the diagnosis stage than in the treatment stage. A previous study concluded that chronic inflammatory conditions have been documented as risk factors for anxiety and depression among cancer patients. The diagnostic phase was found to be associated with a high level of anxiety^43^. Moderate anxiety or depression reported through the corresponding ESAS items (cutoff = 4) can be a useful screening tool for anxiety and depression in non-advanced patients with solid or hematologic malignancies^18^.

Cancer patients with deformities had worse well-being than cancer patients without deformities. Deformity due to malignant disease affects patients' appearance and quality of life, such as oral cancer^44^, Tel Hashomer syndrome, and Guillain‒Barre syndrome in patients with lymphoma^45^. Additionally, poor well-being was reported in smokers, which was supported by other studies^46,47^. In particular, poor socioeconomic status was associated with an increase in almost all severities of the ESAS, which requires more attention to socioeconomic status. Poor quality of life has also been documented in Palestine due to low income^48^.

In terms of quality of life, Palestinian cancer patients face several challenges. For example, the high prevalence of depression in Palestine may be explained by the presence of life stressors, such as siege, occupation^49^, an increased level of anxiety^50^, and difficulties in accessing healthcare^51^. Therefore, palliative care should be included in the healthcare system to improve the quality of life and minimize suffering in these patients. Furthermore, policymakers must integrate specific services, such as palliative care for certain cancer patients, into the health system^52^.

Regarding the strengths of the scales, the ESAS is a practical, patient-centered symptom evaluation instrument that is simple to use, understand, and report. Simultaneous evaluation of ten symptoms enables the identification of symptom clusters and quick assessment. Many clinical and research organizations worldwide use it to benchmark their results. They have psychometrically confirmed their face validity and are available in over 20 languages. It has been determined that there are limited clinically significant differences and responsiveness. It is available in a variety of languages. It is freely available^53^.

### Strengths and limitations

This study included cancer patients from all parts of Palestine, the West Bank, and the Gaza Strip with different socioeconomic statuses. It is also the first study in Palestine documenting depression and palliative care symptoms among oncology patients. However, the current study has several limitations. The major limitation is its cross-sectional design, which does not allow us to see how depression, for example, in cancer patients, changed over time between different treatment paths. Other limitations include the use of convenience sampling from a single tertiary hospital and the use of a small sample size. No stratified analysis was performed for different types of tumors and different treatments.

On the other hand, we have not fully defined all palliative care, as patients may receive care/support not defined in our research. Therefore, the current findings cannot be generalized. Additionally, we use only one scale for assessment, the ESAS, which is a short one-dimensional measure that assesses intensity and severity. Currently, several versions of the ESAS are used, each with a distinct time anchor and several elements, making it impossible to compare or combine the findings. Furthermore, some concepts (for example, well-being) are not clearly defined, and the tenth symptom is different and cannot be unified as headache, constipation, etc.

## Conclusions

The study revealed that fatigue and drowsiness were the most commonly reported moderate to severe symptoms among the cancer patients included in the research. The presence of these symptoms can exert a substantial influence on the daily functioning and overall quality of life experienced by individuals affected by them. A number of sociodemographic and clinical factors were identified as being correlated with distinct symptoms. Factors such as age, gender, marital status, and type of cancer have been identified as influential variables affecting the severity of pain. Likewise, variables such as socioeconomic status, smoking habits, and stage of treatment exhibited associations with the intensity of additional symptoms, encompassing fatigue, somnolence, anxiety, and depression. The research underscores the significance of palliative care in effectively addressing the physical and psychological symptoms encountered by individuals diagnosed with cancer. Palliative care assumes a pivotal role in enhancing the overall quality of life and effectively addressing concurrent ailments such as depression. This study makes a valuable contribution to the expanding corpus of literature on cancer care, with a specific focus on the distinctive obstacles encountered by developing countries. This highlights the necessity for the implementation of comprehensive palliative care services and the development of strategies to effectively address symptom management within these particular contexts.

### Supplementary Information

Supplementary Tables.

## Data Availability

This is an evidence-synthesis study; all data are available from the primary research studies or can be obtained from the corresponding author. Data and materials used in this work are available from the corresponding author upon request. This manuscript forms part of the Master of Community Mental Health Nursing submitted to An-Najah National University, and the abstract was published as part of self-archiving in institutional repositories (that is, university repository: https://repository.najah.edu/items/c1e6d5e3-e9b8-4b0e-8b46-8076da9f91f0).

## Abbreviations

ESAS: Edmonton symptom assessment system
NNUH: An-Najah National University Hospital
QOL: Quality of life
SOB: Shortness of breath
SF-36: 36-Item survey form
BMT: Bone marrow transplantation
AutoBMT: Autologous bone marrow transplant
STAI: State transit anxiety inventory
MOH: Ministry of health
USA: United States of America
ALL: Acute lymphoblastic leukemia
AML: Acute myeloid leukemia
CLL: Chronic lymphocytic leukemia
HL: Hodgkin lymphoma
NHL: Non-Hodgkin lymphoma
MM: Multiple myeloma
MDS: Myelodysplastic syndromes
ESRD: End-stage renal disease
PCBS: Palestinian central bureau of statistics
