# Supplementary material for: Factors associated with palliative care symptoms in cancer patients in Palestine
Source: Sci Rep. 2023 Sep 27;13:16190. doi: 10.1038/s41598-023-43469-0 (PMC10533841; doi:10.1038/s41598-023-43469-0)
Supplement: Supplementary file 1 — Supplementary Tables. [file 41598_2023_43469_MOESM1_ESM.doc]

**Supplementary file 1: Table S1-S9. Table S1:**Pain Dimension (ESAS). **Table S2:**Fatigue Dimension (ESAS). **Table S3:**Drowsiness Dimension (ESAS). **Table S4:** Nausea Dimension (ESAS). **Table S5:** Lack of Appetite Dimension (ESAS). **Table S6:** Shortness of breath Dimension (ESAS). **Table S7:** Depression Dimension (ESAS). **Table S8:** Anxiety Dimension (ESAS). **Table S9:** Poor Feeling of Well-being Dimension (ESAS).

**Table S1** Pain Dimension (ESAS)

| **Variable** | **Pain**  **Median [Q1-Q3]** | **P-value** |
| --- | --- | --- |
| **Age** | | **0.003*** |
| ≤ 50 | 3.0 [1.0-5.0] |
| > 50 | 4.0 [2.0-7.0] |
| **Gender** | | **0.007*** |
| Male | 3.0 [1.0-5.0] |
| Female | 4.0 [2.0-7.0] |
| **Marital status** | | **0.001*** |
| Single | 3.0 [0.0-5.0] |
| Married | 4.0 [2.0-7.0] |
| **Educational level** | | 0.301 |
| School | 4.0 [1.0-6.0] |
| University or college | 4.0 [1.0-6.0] |
| **Socioeconomic status** | | 0.053 |
| Affordable (low) | 4.0 [2.0-6.0] |
| Good (middle) | 4.0 [1.0-5.85] |
| Very Good (high) | 3.0 [0.0-5.5] |
| **Deformities** | | 0.458 |
| Yes | 4.0 [2.5-7.0] |
| No | 4.0 [1.0-6.0] |
| **Smoker** | | 0.17 |
| Yes | 4.0 [2.0-6.0] |
| No | 4.0 [1.0-6.0] |
| **Work** | | **0.001*** |
| Yes | 3.0 [0.0-5.0] |
| No | 4.0 [2.0-7.0] |
| **Living location** | | 0.659 |
| City | 4.0 [1.0-5.0] |
| Village | 4.0 [1.0-7.0] |
| Camp or refugee | 4.0 [2.0-6.0] |
| **Hospitalization status** | | 0.786 |
| Inpatient | 4.0 [1.0-6.0] |  |
| Outpatient | 4.0 [1.0-6.0] |  |
| **Type of cancer** | | **< 0.001*** |
| Hematology | 3.0 [1.0-5.0] |
| Solid | 5.0 [2.0-7.3] |
| **Treatment stage** | | **0.039*** |
| Yes | 4.0[1.0-6.0] |
| No | 3.0 [0.0-5.0] |
| **Currently on chemotherapy** | | **0.036*** |
| Yes | 4.0 [2.0-6.0] |
| No | 3.0 [0.7-5.0] |
| **Recently pancytopenia** | | **0.011*** |
| Yes | 3.0 [1.0-5.0] |
| No | 4.0 [1.5-7.0] |
| **Auto-BMT** | | 0.505 |
| Yes | 4.0 [2.3-6.0] |
| No | 4.0 [1.0-6.0] |
| **Admitted for surgery** | | 0.232 |
| Yes | 4.5 [2.0-8.5] |
| No | 4.0 [1.0-6.0] |
| **Types of psychological support** | | |
| Family support | Yes 4.0 [1.0-6.0]  No 4.0 [1.5-6.0] | 0.456 |
| Social support | Yes 4.0 [2.0-7.0]  No 4.0 [1.0-5.3] | 0.133 |
| Religious support | Yes 4.0 [1.0-7.0]  No 4.0 [1.0-5.0] | 0.376 |
| Health care team support | Yes 4.0 [2.0-7.0]  No 4.0 [1.0-5.0] | 0.1 |

**Table S2** Fatigue Dimension (ESAS)

| **Variable** | **Fatigue**  **Median [Q1-Q3]** | **P-value** |
| --- | --- | --- |
| **Age** | | 0.069 |
| ≤ 50 | 4.0 [2.0-6.5] |
| > 50 | 5.0 [3.0-7.0] |
| **Gender** | | 0.164 |
| Male | 4.0 [2.0-6.0] |
| Female | 5.0 [3.0-7.8] |
| **Marital status** | | **0.006*** |
| Single | 4.0 [2.0-5.0] |
| Married | 5.0 [3.0-7.0] |
| **Educational level** | | 0.262 |
| School | 5.0 [3.0-7.0] |
| University or college | 4.0 [1.0-7.0] |
| **Socioeconomic status** | | **0.053*** |
| Affordable (low) | 5.0 [3.0-7.0] |
| Good (middle) | 4.0 [2.0-6.0] |
| Very Good (high) | 3.0 [0.0-5.5] |
| **Deformities** | | 0.213 |
| Yes | 6.0 [4.0-7.0] |
| No | 5.0 [2.0-7.0] |
| **Smoker** | | 0.164 |
| Yes | 5.0 [3.0-7.0] |
| No | 4.0 [2.0-7.0] |
| **Work** | | **0.042*** |
| Yes | 4.0 [1.0-6.0] |
| No | 5.0 [3.0-7.8] |
| **Living location** | | 0.729 |
| City | 4.0 [2.0-7.0] |
| Village | 5.0 [3.0-7.0] |
| Camp or refugee | 4.0 [2.0-8.0] |
| **Hospitalization status** | | 0.487 |
| Inpatient | 5.0 [2.0-7.0] |
| Outpatient | 5.0 [2.0-6.5] |
| **Type of cancer** | | **0.021*** |
| Hematology | 4.0 [2.0-6.0] |
| Solid | 5.0 [2.7-7.0] |
| **Treatment stage** | | 0.236 |
| Yes | 5.0 [2.5-7.0] |
| No | 4.0 [1.0-7.0] |
| **Currently on chemotherapy** | | 0.185 |
| Yes | 5.0 [3.0-7.0] |
| No | 4.0 [2.0-6.0] |
| **Recently pancytopenia** | | 0.212 |
| Yes | 4.0 [2.0-6.0] |
| No | 5.0 [3.0-7.0] |
| **Auto-BMT** | | 0.463 |
| Yes | 5.0 [3.0-6.8] |
| No | 5.0 [2.0-7.0] |
| **Admitted for surgery** | | 0.335 |
| Yes | 6.0 [2.5-7.5] |
| No | 5.0 [2.0-7.0] |
| **Types of psychological support** | |  |
| Family support | Yes 5.0 [2.0-7.0]  No 5.0 [3.0-7.0] | 0.546 |
| Social support | Yes 5.0 [2.5-8.0]  No 4.0 [2.0-6.0] | 0.115 |
| Religious support | Yes 5.0 [2.0-8.0]  No 4.0[2.3-6.0] | 0.116 |
| Health care team support | **Yes 5.0 [3.0-7.0]**  **No 4.0 [2.0-6.0]** | 0.053 |

**Table S3** Drowsiness Dimension (ESAS)

| **Variable** | **Drowsiness**  **Median [Q1-Q3]** | **P-value** |
| --- | --- | --- |
| **Age** | | 0.379 |
| ≤ 50 | 4.0 [1.5-7.0] |
| > 50 | 5.0 [3.0-7.0] |
| **Gender** | | 0.219 |
| Male | 4.0 [1.0-7.0] |
| Female | 5.0 [2.3-7.0] |
| **Marital status** | | 0.333 |
| Single | 4.0 [2.0-6.0] |
| Married | 5.0 [2.0-7.0] |
| **Educational level** | | 0.630 |
| School | 5.0 [3.0-7.0] |
| University or college | 4.5 [1.3-7.0] |
| **Socioeconomic status** | | **0.002*** |
| Affordable (low) | 5.0 [3.0-7.0] |
| Good (middle) | 4.0 [1.3-6.0] |
| Very Good (high) | 3.0 [0.0-4.5] |
| **Deformities** | | 0.229 |
| Yes | 5.0 [4.0-7.5] |
| No | 5.0 [2.0-7.0] |
| **Smoker** | | 0.085 |
| Yes | 5.0 [3.0-8.0] |
| No | 4.0 [2.0-7.0] |
| **Work** | | 0.075 |
| Yes | 4.0 [1.0-6.0] |
| No | 5.0 [2.3-7.0] |
| **Living location** | | 0.756 |
| City | 4.0 [2.0-7.0] |
| Village | 5.0 [2.0-7.0] |
| Camp or refugee | 5.0 [2.0-7.0] |
| **Hospitalization status** | | 0.442 |
| Inpatient | 4.5 [2.0-7.0] |
| Outpatient | 5.0 [ 2.0-6.5] |
| **Type of cancer** | | 0.122 |
| Hematology | 4.0 [2.0-6.0] |
| Solid | 5.0 [2.0-7.0] |
| **Treatment stage** | | 0.545 |
| Yes | 4.0 [2.0-7.0] |
| No | 5.0 [2.5-6.3] |
| **Currently on chemotherapy** | | 0.522 |
| Yes | 5.0 [2.0-7.0] |
| No | 4.5 [2.0-6.0] |
| **Recently pancytopenia** | | 0.743 |
| Yes | 4.0 [2.0-6.0] |
| No | 5.0 [2.0-7.0] |
| **Auto-BMT** | | 0.778 |
| Yes | 4.5 [3.0-7.8] |
| No | 5.5 [2.0-7.0] |
| **Admitted for surgery** | | 0.778 |
| Yes | 4.5 [1.0-8.5] |
| No | 5.0 [2.0-7.0] |
| **Types of psychological support** | | |
| Family support | Yes 5.0 [2.0-7.0]  No 5.0 [2.0-7.0] | 0.955 |
| Social support | Yes 5.0 [2.0-7.5]  No 4.0 [2.0-6.0] | 0.06 |
| Religious support | Yes 5.0 [2.0-7.0]  No 4.0 [2.0-6.0] | 0.155 |
| Health care team support | Yes 5.0 [2.3-7.0]  No 4.0 [2.0-7.0] | 0.207 |

**Table S4** Nausea Dimension (ESAS)

| **Variable** | **Nausea**  **Median [Q1-Q3]** | **P-value** |
| --- | --- | --- |
| **Age** | | 0.835 |
| ≤ 50 | 2.0 [0.0-5.0] |
| > 50 | 2.5 [1.0-5.0] |
| **Gender** | | 0.795 |
| Male | 2.0 [1.0-5.0] |
| Female | 3.0 [0.0-5.0] |
| **Marital status** | | 0.142 |
| Single | 2.0 [0.0-4.0] |
| Married | 3.0 [1.0-5.0] |
| **Educational level** | | 0.280 |
| School | 3.0 [1.0-5.0] |
| University or college | 1.5 [0.0-5.0] |
| **Socioeconomic status** | | **0.007*** |
| Affordable (low) | 3.0 [1.0-5.0] |
| Good (middle) | 2.0 [0.3-5.0] |
| Very Good (high) | 1.0 [0.0-2.0] |
| **Deformities** | | 0.762 |
| Yes | 3.0 [0.0-4.0] |
| No | 2.0 [0.7-5.0] |
| **Smoker** | | 0.152 |
| Yes | 2.5 [1.0-6.0] |
| No | 2.0 [0.0-5.0] |
| **Work** | | 0.602 |
| Yes | 2.0 [1.0-4.0] |
| No | 3.0 [0.0-5.0] |
| **Living location** | | 0.312 |
| City | 2.0 [0.0-4.0] |
| Village | 3.0 [0.5-5.0] |
| Camp or refugee | 3.0 [1.0-7.0] |
| **Hospitalization status** | | 0.213 |
| Inpatient | 3.0 [0.0-5.0] |
| Outpatient | 2.0 [1.0-5.0] |
| **Type of cancer** | | 0.562 |
| Hematology | 2.0 [0.0-5.0] |
| Solid | 3.0 [1.0-5.0] |
| **Treatment stage** | | 0.719 |
| Yes | 2.0 [1.0-5.0] |
| No | 1.5 [0.0-5.3] |
| **Currently on chemotherapy** | | **0.023*** |
| Yes | 3.0 [1.0-5.0] |
| No | 1.0 [0.0-4.0] |
| **Recently pancytopenia** | | 0.304 |
| Yes | 2.0 [0.0-5.0] |
| No | 3.0 [1.0-5.0] |
| **Auto-BMT** | | 0.228 |
| Yes | 3.0 [1.0-7.0] |
| No | 2.0 [0.0-5.0] |
| **Admitted for surgery** | | 0.887 |
| Yes | 2.0 [0.8-4.5] |
| No | 2.0 [0.0-5.0] |
| **Types of psychological support** | | |
| Family support | Yes 2.5 [0.8-5.0]  No 2.0 [0.0-5.0] | 0.849 |
| Social support | Yes 3.0 [1.0-5.]  No 2.0 [0.0-4.0] | 0.201 |
| Religious support | Yes 3.0 [1.0-5.0]  No 2.0 [0.0-5.0] | 0.375 |
| Health care team support | Yes 2.0 [1.0-5.0]  No 2.0 [0.0-5.0] | 0.549 |

**Table S5 Lack of Appetite Dimension (ESAS)**

| **Variable** | **Lack of appetite**  **Median [Q1-Q3]** | **P-value** |
| --- | --- | --- |
| **Age** | | 0.098 |
| ≤ 50 | 4.0 [1.0-6.0] |
| > 50 | 4.0 [1.0-7.0] |
| **Gender** | | 0.728 |
| Male | 4.0 [1.0-7.0] |
| Female | 4.0 [1.0-7.0] |
| **Marital status** | | **0.036*** |
| Single | 3.0 [1.0-6.0] |
| Married | 4.0 [1.0-7.0] |
| **Educational level** | | 0.109 |
| School | 4.0 [1.0-7.0] |
| University or college | 3.0 [1.0-6.8] |
| **Socioeconomic status** | | **0.004*** |
| Affordable (low) | 4.0 [2.0-7.0] |
| Good (middle) | 4.0 [1.0-6.0] |
| Very Good (high) | 1.0 [0.0-4.5] |
| **Deformities** | | 0.798 |
| Yes | 4.0 [0.5-5.5] |
| No | 4.0 [1.0-7.0] |
| **Smoker** | | **0.016*** |
| Yes | 4.5 [2.0-8.0] |
| No | 4.0 [1.0-6.0] |
| **Work** | | **0.027*** |
| Yes | 3.0 [1.0-5.0] |
| No | 4.0 [1.0-7.0] |
| **Living location** | | 0.681 |
| City | 4.0 [1.0-7.0] |
| Village | 4.0 [1.0-7.0] |
| Camp or refugee | 3.0 [1.0-6.0] |
| **Hospitalization status** | | **0.007*** |
| Inpatient | 4.5 [1.0-8.0] |
| Outpatient | 3.0 [1.0-5.5] |
| **Type of cancer** | | 0.724 |
| Hematology | 4.0 [1.0-7.0] |
| Solid | 4.0 [1.0-6.3] |
| **Treatment stage** | | 0.713 |
| Yes | 4.0 [1.0-7.0] |
| No | 4.0 [0.0-7.0] |
| **Currently on chemotherapy** | | 0.094 |
| Yes | 4.0 [1.0-7.0] |
| No | 3.0 [1.0-5.0] |
| **Recently pancytopenia** | | 0.687 |
| Yes | 4.5 [1.0-7.0] |
| No | 4.0 [1.0-6.0] |
| **Auto-BMT** | | **0.037*** |
| Yes | 6.0 [1.3-9.5] |
| No | 4.0 [1.0-7.0] |
| **Admitted for surgery** | | 0.493 |
| Yes | 2.5 [1.0-5.0] |
| No | 4.0 [1.0-7.0] |
| **Types of psychological support** |  |
| Family support | Yes 4.0 [1.0-7.0]  No 4.0 [1.0-7.0] | 0.359 |
| Social support | Yes 4.0 [1.0-7.0]  No 4.0 [1.0-7.0] | 0.726 |
| Religious support | Yes 3.0 [1.0-6.0]  No 4.0 [1.0-7.0 | 0.517 |
| Health care team support | Yes 4.0 [1.0-7.0]  No 4.0 [1.0-7.0] | 0.6 |

**Table S6 Shortness of breath Dimension (ESAS)**

| **Variable** | **SOB**  **Median [Q1-Q3]** | **P-value** |
| --- | --- | --- |
| **Age** | | 0.651 |
| ≤ 50 | 1.0 [0.0-4.0] |
| > 50 | 1.0 [0.0-4.0] |
| **Gender** | | 0.22 |
| Male | 1.0 [0.0-4.0] |
| Female | 1.0 [0.0-4.0] |
| **Marital status** | | 0.461 |
| Single | 1.0 [0.0-4.0] |
| Married | 1.0 [0.0-4.0] |
| **Educational level** | | 0.123 |
| School | 1.0 [0.0-4.0] |
| University or college | 0.0 [0.0-4.0] |
| **Socioeconomic status** | | 0.114 |
| Affordable (low) | 1.0 [0.0-4.0] |
| Good (middle) | 1.0 [0.0-5.0] |
| Very Good (high) | 0.0 [0.0-2.0] |
| **Deformities** | | 0.264 |
| Yes | 3.0 [0.0-5.5] |
| No | 1.0 [0.0-4.0] |
| **Smoker** | | **0.021*** |
| Yes | 2.5 [0.0-5.0] |
| No | 1.0 [0.0-3.0] |
| **Work** | | 0.145 |
| Yes | 1.0 [0.0-4.0] |
| No | 1.0 [0.0-4.0] |
| **Living location** | | 0.361 |
| City | 1.0 [0.0-4.0] |
| Village | 1.0 [0.0-4.0] |
| Camp or refugee | 2.0 [0.0-5.0] |
| **Hospitalization status** | | 0.088 |
| Inpatient | 1.0 [0.0-4.0] |
| Outpatient | 1.0 [0.0-4.0] |
| **Type of cancer** | | 0.553 |
| Hematology | 1.0 [0.0-4.0] |
| Solid | 1.0 [0.0-4.0] |
| **Treatment stage** | | 0.81 |
| Yes | 1.0 [0.0-4.0] |
| No | 1.5 [0.0-5.0] |
| **Currently on chemotherapy** | | 0.082 |
| Yes | 1.0 [0.0-4.0] |
| No | 0.5 [0.0-3.0] |
| **Recently pancytopenia** | | 0.231 |
| Yes | 1.5 [0.0-4.0] |
| No | 1.0 [1.0-4.0] |
| **Auto-BMT** | | 0.672 |
| Yes | 1.5 [0.0-4.8] |
| No | 1.0 [0.0-4.0] |
| **Admitted for surgery** | | 0.729 |
| Yes | 1.5 [0.8-2.0] |
| No | 1.0 [0.0-4.0] |
| **Types of psychological support** | |  |
| Family support | Yes 1.0 [0.0-4.0]  No 2.0 [0.0-5.0] | **0.034*** |
| Social support | Yes 1.0 [0.0-4.0]  No 1.0 [0.0-4.0] | 0.86 |
| Religious support | Yes 1.0 [0.0-4.0]  No 1.0 [0.0-4.0] | 0.511 |
| Health care team support | Yes 1.0 [0.0-4.0]  No 1.0 [0.0-4.0] | 0.505 |

**Table S7** Depression Dimension (ESAS)

| **Variable** | **Depression**  **Median [Q1-Q3]** | **P-value** |
| --- | --- | --- |
| **Age** | | 0.232 |
| ≤ 50 | 3.0 [1.0-5.0] |
| > 50 | 2.0 [1.0-5.0] |
| **Gender** | | 0.82 |
| Male | 2.0 [1.0-5.0] |
| Female | 3.0 [1.0-5.0] |
| **Marital status** | | 0.599 |
| Single | 3.0 [1.0-4.0] |
| Married | 2.0 [1.0-5.0] |
| **Educational level** | | 0.151 |
| School | 3.0 [1.0-5.0] |
| University or college | 2.0 [0.0-5.0] |
| **Socioeconomic status** | | **0.026*** |
| Affordable (low) | 3.0 [1.0-3.3] |
| Good (middle) | 2.0 [1.0-5.0] |
| Very Good (high) | 1.0 [0.0-3.5] |
| **Deformities** | | 0.441 |
| Yes | 4.0 [0.0-6.5] |
| No | 2.0 [1.0-5.0] |
| **Smoker** | | **0.004*** |
| Yes | 4.0 [1.0-6.0] |
| No | 2.0 [1.0-4.0] |
| **Work** | | 0.193 |
| Yes | 2.0 [1.0-4.0] |
| No | 3.0 [1.0-5.0] |
| **Living location** | | 0.213 |
| City | 3.0 [1.0-5.0] |
| Village | 2.0 [1.0-5.0] |
| Camp or refugee | 4.0 [0.0-6.0] |
| **Hospitalization status** | | 0.12 |
| Inpatient | 3.0 [1.0-6.0] |
| Outpatient | 2.0 [1.0-4.5] |
| **Type of cancer** | | 0.739 |
| Hematology | 2.0 [0.5-5.0] |
| Solid | 3.0 [1.0-5.0] |
| **Treatment stage** | | 0.375 |
| Yes | 3.0 [1.0-5.0] |
| No | 2.0 [0.0-4.0] |
| **Currently on chemotherapy** | | 0.503 |
| Yes | 3.0 [1.0-5.0] |
| No | 2.0 [1.0-4.3] |
| **Recently pancytopenia** | | 0.444 |
| Yes | 3.0 [1.0-6.0] |
| No | 2.0 [1.0-5.0] |
| **Auto-BMT** | | 0.219 |
| Yes | 3.0 [1.0-6.8] |
| No | 2.0 [1.0-5.0] |
| **Admitted for surgery** | | 0.7 |
| Yes | 3.5 [0.0-6.0] |
| No | 2.0 [1.0-5.0] |
| **Types of psychological support** | |  |
| Family support | Yes 2.0 [1.0-5.0]  No 3.0 [1.0-5.5] | 0.124 |
| Social support | Yes 2.0 [1.0-4.5]  No 3.0 [1.0-5.0] | 0.498 |
| Religious support | Yes 2.0 [1.0-5.0]  No 3.0 [0.3-5.0] | 0.851 |
| Health care team support | Yes 2.5 [1.0-5.0  No 2.0 [0.0-5.0] | 0.669 |

**Table S8** Anxiety Dimension (ESAS)

| **Variable** | **Anxiety**  **Median [Q1-Q3]** | **P-value** |
| --- | --- | --- |
| **Age** | | 0.242 |
| ≤ 50 | 4.0 [1.0-7.0] |
| > 50 | 3.0 [1.0-6.0] |
| **Gender** | | 0.107 |
| Male | 3.0 [1.0-6.0] |
| Female | 4.0 [1.0-7.0] |
| **Marital status** | | 0.607 |
| Single | 3.0 [1.0-7.0] |
| Married | 3.0 [1.0-6.0] |
| **Educational level** | | **0.044*** |
| School | 4.0 [1.0-7.0] |
| University or college | 2.0 [1.0-6.0] |
| **Socioeconomic status** | | **0.012*** |
| Affordable (low) | 4.0 [1.0-7.0] |
| Good (middle) | 3.0 [1.0-5.0] |
| Very Good (high) | 2.0 [0.5-4.5] |
| **Deformities** | | 0.258 |
| Yes | 6.0 [1.5-7.5] |
| No | 3.0 [1.0-6.0] |
| **Smoker** | | 0.056 |
| Yes | 4.0 [1.0-7.0] |
| No | 3.0 [1.0-6.0] |
| **Work** | | 0.296 |
| Yes | 3.0 [1.0-6.0] |
| No | 3.5 [1.0-7.0] |
| **Living location** | | 0.381 |
| City | 3.0 [1.0-6.0] |
| Village | 3.0 [1.0-6.0] |
| Camp or refugee | 4.0 [2.0-8.0] |
| **Hospitalization status** | | 0.14 |
| Inpatient | 4.0 [1.0-7.0] |
| Outpatient | 3.0 [1.0-5.5] |
| **Type of cancer** | | 0.208 |
| Hematology | 3.0 [1.0-7.0] |
| Solid | 3.0 [1.0-5.0] |
| **Treatment stage** | | **< 0.001*** |
| Yes | 3.0 [1.0-6.0] |
| No | 5.0 [3.8-9.3] |
| **Currently on chemotherapy** | | 0.353 |
| Yes | 3.0 [1.0-6.0] |
| No | 3.0 [0.8-6.3] |
| **Recently pancytopenia** | | 0.148 |
| Yes | 3.5 [1.0-7.0] |
| No | 3.0 [1.0-6.0] |
| **Auto-BMT** | | 0.544 |
| Yes | 3.0 [1.0-8.0] |
| No | 3.0 [1.0-6.0] |
| **Admitted for surgery** | | 0.208 |
| Yes | 5.5 [0.8-10.0] |
| No | 3.0 [1.0-6.0] |
| **Types of psychological support** | |  |
| Family support | Yes 3.0 [1.0-6.0]  No 4.0 [1.0-7.0] | 0.195 |
| Social support | Yes 3.0 [1.0-6.0]  No 3.0 [1.0-7.0] | 0.696 |
| Religious support | Yes 3.0 [1.0-6.0]  No 3.0 [1.0-6.0] | 0.713 |
| Health care team support | Yes 3.0 [1.0-6.0]  No 3.0 [1.0-6.0] | 0.359 |

**Table S9** Poor Feeling of Well-being Dimension (ESAS)

| **Variable** | **Well-being***  **Median [Q1-Q3]** | **P-value** |
| --- | --- | --- |
| **Age** | | 0.306 |
| ≤ 50 | 4.0 [1.0-6.0] |
| > 50 | 4.0 [1.0-6.0] |
| **Gender** | | 0.11 |
| Male | 4.0 [1.0-5.0] |
| Female | 4.0 [1.0-7.0] |
| **Marital status** | | 0.104 |
| Single | 3.0 [1.0-6.0] |
| Married | 0.154 |
| **Educational level** | | 0.47 |
| School | 4.0 [1.0-7.0] |
| University or college | 3.0 [1.0-5.8] |
| **Socioeconomic status** | | **0.016*** |
| Affordable (low) | 4.0 [1.0-7.0] |
| Good (middle) | 4.0 1.0-5.0] |
| Very Good (high) | 2.0 [0.0-4.0] |
| **Deformities** | | **0.026*** |
| Yes | 5.0 [3.5-9.5] |
| No | 4.0 [1.0-6.0] |
| **Smoker** | | **0.028*** |
| Yes | 5.0 [1.3-7.0]  4.0 [1.0-6.0] |
| No |
| **Work** | | 0.05 |
| Yes | 3.0 [1.0-5.0] |
| No | 4.0 [1.0-6.8] |
| **Living location** | | 0.47 |
| City | 4.0 [1.0-6.0] |
| Village | 4.0 [1.0-6.0] |
| Camp or refugee | 5.0 [1.0-8.0] |
| **Hospitalization status** | | 0.169 |
| Inpatient | 4.0 [1.0-7.0] |
| Outpatient | 4.0 [1.0-6.0] |
| **Type of cancer** | | 0.938 |
| Hematology | 4.0 [1.0-6.0] |
| Solid | 4.0 [1.0-6.0] |
| **Treatment stage** | | 0.81 |
| Yes | 4.0 [1.0-6.0] |
| No | 4.0 [0.0-8.0] |
| **Currently on chemotherapy** | | 0.125 |
| Yes | 4.0 [1.0-6.] |
| No | 3.0 [0.8-5.3] |
| **Recently pancytopenia** | | 0.319 |
| Yes | 4.0[0.7-6.0] |
| No | 4.0[1.0-6.0] |
| **Auto-BMT** | | 0.736 |
| Yes | 4.0 [2.0-5.8] |
| No | 4.0 [1.0-6.0] |
| **Admitted for surgery** | | 0.899 |
| Yes | 4.5 [1.0-5.5] |
| No | 4.0 [1.0-6.0] |
| **Types of psychological support** | |  |
| Family support | Yes 4.0 [1.0-6.0]  No 4.0 [2.0-6.0] | 0.096 |
| Social support | Yes 4.0 [1.0-6.5]  No 4.0 [1.0-6.0] | 0.866 |
| Religious support | Yes 4.0 [1.0-6.0]  No 4.0 [1.0-6.0] | 0.553 |
| Health care team support | Yes 4.0 [1.0-6.0]  No 4.0 [1.0-6.0] | 0.632 |
